# Comparison of Thessaly Test with Joint Line Tenderness and McMurray Test in the Diagnosis of Meniscal Tears

**DOI:** 10.5704/MOJ.2007.018

**Published:** 2020-07

**Authors:** B Shekarchi, A Panahi, SA Raeissadat, N Maleki, S Nayebabbas, P Farhadi

**Affiliations:** 1 Department of Radiology, AJA University of Medical Sciences, Tehran, Iran; 2 Chronic Respiratory Diseases Research Center, National Research Institute of Tuberculosis and Lung Diseases (NRITLD), Tehran, Iran; 3 Department of Physical Medicine and Rehabilitation, Shaheed Beheshti University of Medical Sciences, Tehran, Iran; 4 Department of Physical Medicine and Rehabilitation, AJA University of Medical Sciences, Tehran, Iran; 5 Department of Radiology, Shiraz University of Medical Sciences, Shiraz, Iran

**Keywords:** Thessaly, joint line tenderness, McMurray, meniscal tears

## Abstract

**Introduction:**

Meniscus injuries are the most frequent problem of the knee. The aim of this study was to investigate the accuracy of the Thessaly test and comparing it with those of McMurray and Joint-line tenderness tests for diagnosing meniscal tears.

**Materials and methods:** This study was designed as a prospective observational one done in an outpatient clinic at a university hospital. 106 patients with knee pain and 82 age-matched control were included during study period (from February 2014 to January 2015). Each patient was clinically examined with McMurray, Thessaly, and joint line tenderness tests. Then, the findings were matched by MRI and arthroscopic findings. The sensitivity, specificity, positive predictive value, negative predictive value and accuracy were calculated as main outcomes.

**Results:**

Based on MRI, Thessaly was the most sensitive for medial meniscus tears (56.2%), while McMurray and joint-line tenderness were more specific (89.1% and 88.0%, respectively). For lateral meniscus tears, McMurray was the most sensitive (56.2%) and all were specific (McMurray 89.6%, Thessaly 88.4%, joint-line tenderness 90.2%). With arthroscopy, Thessaly was the most sensitive for medial meniscus (76.6%), while McMurray and joint-line tenderness were more specific (81.0%, and 81.0%). Agreement with arthroscopy was the highest with McMurray (for medial meniscus kappa=0.40, p<0.001, and for lateral meniscus kappa=0.38, p=0.002).

**Conclusion:**

The Thessaly can be used to screen for medial meniscus tears. McMurray and joint-line tenderness should be used for suspected medial meniscus tears. For lateral meniscus, McMurray is appropriate for screening and all the tests are useful in clinic.

## Introduction

Knee pain comprises approximately one third of all outpatient physical therapy visits^1^ of which 9% are associated with meniscal lesions^2^. A combination of subjective symptoms increases the predictive value of recognising meniscal lesion to 70-80%, but patient history is not enough for accurate diagnosis^3^.

Various physical tests have been described in the literature: the McMurray’s, the Apley compression and distraction, and the joint line tenderness test with their adaptations are known examples^4-14^. The diagnostic accuracy of the clinical tests may improve with compressive forces applied to the knee joint with weight-bearing axial compression^15, 16^. Medial joint line tenderness, knee locking, daily pain and work absence have been reported to predict 61% of meniscal tear confirmed by arthroscopy^3^.

More recently, the Thessaly test was suggested to be safely used as a first line screening test for identifying the candidates of arthroscopic meniscal surgery. The test also was reported to have a lower false positive and false negative compared to other clinical tests^16^. On the other hand, MRI is considered as the diagnostic method of choice because of providing a reliable non-invasive tool in identifying common knee pathology^4, 17^. Arthroscopy is also the gold standard for the diagnosis of meniscal lesions, with 90-95% accuracy^18^. In addition, arthroscopy provides simultaneous therapeutic possibilities.

Low specificity, sensitivity, and diagnostic accuracy of the clinical tests may leave meniscal lesions undetected. The methodological quality of the studies on the clinical tests has a significant effect on reported sensitivities and specificities as well^2, 19^.

High cost and false positives are the disadvantages of MRI for the diagnosis of meniscal lesions^4, 20^. A false positive of 65% for identifying medial meniscal tears and 43% for lateral meniscus tears have been estimated for MRI when compared with surgical findings^21^. Even, some researchers believe that MRI is not superior to physical examination in the diagnosis of meniscal tears^20^. Invasiveness and high cost are the major drawbacks for common use of arthroscopy too^4^.

Therefore, we need less expensive, non-invasive, and more accurate methods of identifying meniscal lesions. The optimal clinical tests and measures should be selected with high sensitivity and specificity. The aim of conducting the present study was to investigate the accuracy of the Thessaly test in screening for meniscal tears. We examined whether the Thessaly test alone or in combination with other clinical tests could provide a non-invasive means of identifying lesions.

## Materials and Methods

The present study was designed as a prospective observational one which was conducted from February 2014 to January 2015 in an outpatient clinic of physical medicine and rehabilitation at the university hospital; Imam Reza, a large referral practice and research centre in Tehran. The study protocol was approved by the institutional review board (IRB) of AJA University of Medical Sciences and we obtained ethics approval from the local ethics committee before the study was commenced. All the participants read and signed an informed consent form prior to study involvement.

Consecutive patients aged more than 15 years who had a current knee pain or discomfort, history of swelling or effusion, knee locking, or clicking sensation on movement of the knee were enrolled in the study. Exclusion criteria included individuals with a previous history of knee surgery or arthroscopy, and the radiologic evidence or clinical manifestations of osteoarthritis and rheumatologic diseases. In addition, we excluded patients if they could not attend the clinical examinations and did not take MRI or refused arthroscopy.

At the first visit and according to predetermined inclusion and exclusion criteria patients were screened for eligibility. From 220 eligible patients, 106 completed the tests and composed our analytic sample. We also selected 100 age-matched participants as the group control from which 82 completed the study. The control group was randomly selected from patients who came to the clinic for complaints other than knee problems (e.g., pain in their shoulders).

At the beginning of the study, research staff was briefed. Our clinical assessors were two specialists of physical medicine and rehabilitation. The study was carried out under the supervision of two board-certified physical medicine and rehabilitation specialists, and an associate professor of radiology.

Patients were interviewed to obtain their past medical histories. The presence of the symptoms and signs of meniscal lesions and other knee problems were questioned. In addition, the recruitment questionnaire asked about consultation with a physician and active or previous treatment and surgery. Then our clinical assessors performed physical examinations and conducted further investigations for the diagnosis of meniscal lesions. We recorded any pain or discomfort, clicking sensation or locking on movement, and swelling and injury of the knee.

For the Thessaly test, participants were instructed to stand while supported by holding their hands. Thereafter, they were guided to perform internal and external rotation of the knee and body in three occasions during which the knee was flexed at almost 20°. We considered the test as positive when the patients had medial or lateral joint-line discomfort or experienced locking in the knee^4^. For all the participants, we performed McMurray and joint line tenderness at the lateral and medial sides. Further investigations including MRI were ordered and lesions were graded^22^. Then, the patients with suspected meniscal lesions were referred to arthroscopy for definitive diagnosis and appropriate treatment.

We used a block design to randomise the patients to the observers. The investigators who performed physical examinations were blinded to patients’ medical history. Moreover, the assigned radiologists also were unaware of the study question and patients’ medical history.

All statistical analyses were performed with the Statistical Package for Social Sciences version 16.0 [SPSS Inc, Chicago, IL, USA]. The distribution of variables was analysed with Kolmogorov-Smirnov test. Continuous data are presented as mean and standard deviation, and categorical data, as numbers and proportions. We used Chisquared and t-test to assess differences in demographic characteristics between the two groups. Sensitivity, specificity, positive and negative predictive values, positive and negative likelihood ratios, the accuracy of diagnoses, and diagnostic odds were calculated by forming two-by-two cross table and using standard formula. Combinations of the tests were also evaluated statistically to find the best diagnostic strategy in the clinic. A p-value of less than 0.05 was considered significant.

## Results

A total of 188 patients were included in the study. The mean age of the patients in was found to be 23.9±5.1 (range 19-44) years, and sex distribution was 115 males (61.2%) and 73 females (38.8%). We performed MRI for all patients. Overall, 96 (51%) participants had medial meniscus tears including 23 (12.2%) grade I, 42 (22.3%) grade II, 27 (14.4%) grade III, and 4 (2.1%) grade IV. Also, 16 (8.5%) patients had lateral meniscus tears including 7 (3.7%) grade I, 3 (1.6%) grade II, and 6 (3.2%) grade III. There was no grade IV lateral meniscus tear among the participants. Other findings on MRI included 63 (33.5%) incomplete and 16 (8.5%) complete anterior cruciate, 2 (1.1%) incomplete posterior cruciate, 36 (19.1%) incomplete medial and lateral collateral, 9 (4.8%) incomplete lateral collateral, and 1 complete lateral collateral ligament tears. In total, 126 (67%) participants had at least one lesion in their menisci or cruciate ligaments, confirmed by MRI. Of these 126 patients, 26 were from the group control. In addition, 7 participants had combined tears of menisci and anterior cruciate ligament. Based on MRI, Thessaly was the most sensitive for medial meniscus tears (56.2%), while McMurray and joint-line tenderness were more specific (89.1% and 88.0%, respectively). For lateral meniscus tears, McMurray was the most sensitive (56.2%) and all were specific (McMurray 89.6%, Thessaly 88.4%, joint-line tenderness 90.2%). Table I, demonstrates the diagnostic parameters based on the results of MRI.

**Table I:** The diagnostic parameters of the three clinical tests with MRI as the standard method of diagnosis (N = 188)

	Diagnosis	
Clinical test	Medial meniscus tear	Lateral meniscus tear
**McMurray**		
Sensitivity	44.8%	56.2%
Specificity	89.1%	89.6%
False positive	10	17
False negative	53	7
Accuracy	66.4%	83.0%
**Joint-line tenderness**		
Sensitivity	43.8%	43.8%
Specificity	88.0%	90.2%
False positive	11	16
False negative	54	9
Accuracy	65.4%	82.4%
**Thessaly**		
Sensitivity	56.2%	50.0%
Specificity	79.3%	88.4%
False positive	19	19
False negative	42	8
Accuracy	67.5%	81.4%
**All the tests combined**		
Sensitivity	66.7%	68.8%
Specificity	73.9%	84.3%
False positive	24	27
False negative	32	5
Accuracy	70.0%	83.0%

As the gold standard, arthroscopy was performed for 68 (36.2%) patients and the findings included 47 (69%) medial and 16 (23%) lateral meniscus tears. In addition, we found 40 (59%) anterior and 1 (1.5%) posterior cruciate ligament tears on arthroscopy. Thessaly was the most sensitive for medial meniscus tears (76.6%), while McMurray and joint-line tenderness were more specific (81.0%, and 81.0%). The most sensitive test for lateral meniscus tears was combined tests with 68.8% sensitivity, while McMurray test had highest specificity (83%). (Table II) demonstrates the diagnostic parameters based on the results of knee arthroscopy. We also measured the agreements between the results of the clinical tests and arthroscopy for the diagnosis of meniscal tears (Table III).

**Table II:** The diagnostic parameters of the three clinical tests with arthroscopy as the gold standard of the diagnosis (N = 68)

	Diagnosis	
Clinical test	Medial meniscus tear	Lateral meniscus tear
**McMurray**		
Sensitivity	66.0%	56.2%
Specificity	81.0%	83.0%
False positive	4	8
False negative	16	7
Accuracy	70.5%	70.5%
Positive predictive value	88.6%	59.2%
Negative predictive value	51.5%	84.8%
Positive likelihood ratio	3.5	3.3
Negative likelihood ratio	0.4	0.5
**Joint-line tenderness**		
Sensitivity	61.7%	50.0%
Specificity	81.0%	78.7%
False positive	4	10
False negative	18	8
Accuracy	67.6%	66.2%
Positive predictive value	87.9%	44.4%
Negative predictive value	48.6%	82.2%
Positive likelihood ratio	3.2	2.3
Negative likelihood ratio	0.5	0.6
**Thessaly**		
Sensitivity	76.6%	50.0%
Specificity	52.4%	74.5%
False positive	10	12
False negative	11	8
Accuracy	69.0%	63.2%
Positive predictive value	78.3%	40.0%
Negative predictive value	50.0%	81.4%
Positive likelihood ratio	1.6	2.0
Negative likelihood ratio	0.4	0.7
**All the tests combined**		
Sensitivity	83.0%	68.8%
Specificity	42.9%	69.2%
False positive	12	16
False negative	8	5
Accuracy	70.5	69.0%
Positive predictive value	76.5%	40.7%
Negative predictive value	52.9%	87.8%
Positive likelihood ratio	1.4	2.2
Negative likelihood ratio	0.4	0.4

**Table III:** Agreements between the results of the clinical tests and arthroscopy

	Clinical tests	Proportion in agreement	Kappa	p-value
Medial meniscus	McMurray	70.6%	0.40	<0.001
	Joint-line Tenderness	67.6%	0.36	0.001
	Thessaly	69.1%	0.29	0.018
Lateral meniscus	McMurray	76.2%	0.38	0.002
	Joint-line Tenderness	71.4%	0.28	0.028
	Thessaly	68.3%	0.23	0.069*
Both menisci	All the tests combined	81.0%	0.16	0.105*

* Non-significant

## Discussion

Meniscal tears are the result of injury or degeneration of the substance of the meniscus. Majority patients with meniscal tears complain of an acute onset of sharp pain following a twisting injury with the knee flexed and the foot planted on the ground^23^. The diagnosis of meniscus tear can frequently be made from a careful history, physical examination and appropriate diagnostic tests. There are various provocative manoeuvres or tests to elicit symptoms from a torn meniscus, but the reported accuracy have been inconsistent across studies. In this study, we tried to compare the accuracy of Thessaly test with those of McMurray and joint line tenderness in screening for meniscal tears and to examine whether the tests alone or combined could provide a non-invasive means of identifying lesions. We compared the results of the clinical tests with findings of MRI as a non-invasive method, and of arthroscopy as the diagnostic gold standard.

Our results demonstrated that when MRI is the basis for the diagnosis of medial meniscus tears, Thessaly is the most sensitive, while McMurray and joint-line tenderness are more specific clinical tests. In other words, a negative Thessaly test is more helpful in ruling out medial meniscus tears and a positive McMurray or joint-line tenderness test can be used for ruling in the tears. For lateral meniscus tears McMurray is the most sensitive and all the three tests are highly specific.

With arthroscopy the results again showed that for the diagnosis of medial meniscus tears Thessaly is the most sensitive while McMurray and joint-line tenderness are more specific clinical tests. The three tests seem to be equally accurate for the diagnosis of medial meniscus tears. Positive predictive value is the largest for McMurray, while negative predictive value is almost the same for the three tests. In addition, McMurray test possesses the highest positive likelihood ratio. For lateral meniscus tears McMurray is the most sensitive, specific, and accurate test with the largest positive predictive value and likelihood ratio. For tears in anterior cruciate ligament, medial and lateral menisci, together, joint-line tenderness is the most sensitive and McMurray is the most specific. Agreement between the result of the clinical tests and arthroscopy was the highest for McMurray test.

In a study by Gupta *et al*^24^ with 66 knees to validate joint line tenderness, the sensitivity, specificity, positive predictive value, negative predictive value and accuracy in diagnosing medial meniscus tears found to be 50%, 61.7%, 51.8%, 60% and 56.4%, respectively. The sensitivity, specificity, positive predictive value, negative predictive value and accuracy for McMurray's test for diagnosing medial meniscus tear were 54%, 79%, 68% , 67.50% and 67.74%, respectively. The author concluded that clinical tests like McMurray and joint line tenderness have low diagnostic value when applied individually. They may be useful when combined together with the background of clinical history. The diagnostic parameters of McMurray test was in concordance with our results, while those were lower than our study regarding joint line tenderness.

In a study by Karachalios *et al*^16^, 213 patients with knee injuries underwent MRI and arthroscopic surgery, and 197 asymptomatic participants underwent MRI on their normal knees. It was reported that the Thessaly test at 20° of knee flexion had a diagnostic accuracy of 94% for the detection of medial meniscus tears and 96% for the lateral meniscus tears, and that the test had a low rate of false-positive and false-negative recordings. They suggested that other clinical tests showed inferior accuracies, except for joint line tenderness with the accuracy of 89% in the detection of lateral meniscal tears. It was concluded that the Thessaly test at 20° of knee flexion can be used effectively as a first-line clinical screening test. They even claimed that the test will reduce the need for and the cost of modern MRI methods. Our results failed to show the superiority of the Thessaly test. In another study on 109 (80 males) patients with average age of 39 (16 to 56) years, the diagnostic accuracy of the Thessaly test was compared with joint line tenderness and McMurray’s tests. Patients were presented with a history suggestive of a meniscal tear. They underwent MRI and arthroscopy. The study showed a relatively low diagnostic accuracy for the Thessaly (61% for medial meniscus and 80% for lateral meniscus), and the McMurray’s tests (57% for medial meniscus and 77% for lateral meniscus). It was declared that the joint line tenderness test is the most accurate (81% for medial meniscus and 90% for lateral meniscus). Also, it was suggested that combining the tests further increased the accuracy. It was concluded that the Thessaly test in isolation was not useful for the detection of meniscal tears but it helps to increase diagnostic certainty when combined with other tests^4^. With regard to the Thessaly test and when MRI was considered as the standard diagnostic method, the results of that study were comparable to ours. However, we calculated similar accuracies for both menisci, when arthroscopy was used as the gold standard. Other similarities can be recognised between the two studies for the McMurray test, especially when MRI is considered as the basis for the comparisons. But, our results did not support the concept of superiority for the joint line tenderness test, specifically for arthroscopy as the gold standard. It should be noted that there are other discrepancies between the two studies. For example, we selected participants randomly, and used a control group to provide higher methodological quality.

Our sample was sufficiently large to detect important differences among the diagnostic parameters. Our research team and assessors were highly trained, and they attempted to follow diagnostic protocols strictly. Meanwhile, ethical considerations prevent us to perform arthroscopy when it was not necessary. Therefore, we were not able to compare the clinical tests with the gold standard for all participants.

## Conclusion

Our study showed that the Thessaly is a sensitive clinical test for the diagnosis of medial meniscus tears. Therefore, the test can be used to screen general population. On the other hand, McMurray and joint-line tenderness are more specific and should be used to diagnose medial meniscus tears in patients with the related clinical manifestations. For lateral meniscus tears, the McMurray test is appropriate for screening and all the three tests are useful in the physical examination of patients with the suspected tears. Therefore, with regard to sensitivity, specificity, and accuracy, the Thessaly should be used along with the other tests. Overall, we did not find important difference in the diagnostic usefulness of the three tests and prefer to use the tests combined. The tests combined have a high proportion in agreement with arthroscopy and are valuable in the detection of meniscal and the cruciate ligament lesions.
